# Hybrid magnetite nanoparticles/*Rosmarinus officinalis *essential oil nanobiosystem with antibiofilm activity

**DOI:** 10.1186/1556-276X-7-209

**Published:** 2012-04-10

**Authors:** Carmen Chifiriuc, Valentina Grumezescu, Alexandru Mihai Grumezescu, Crina Saviuc, Veronica Lazăr, Ecaterina Andronescu

**Affiliations:** 1 Faculty of Biology, University of Bucharest, Bucharest, Romania; 2 Faculty of Applied Chemistry and Materials Science, Politechnica University of Bucharest, Bucharest, Romania

## Abstract

Biofilms formed by fungal organisms are associated with drastically enhanced resistance against most antimicrobial agents, contributing to the persistence of the fungi despite antifungal therapy. The purpose of this study is to combine the unique properties of nanoparticles with the antimicrobial activity of the *Rosmarinus officinalis *essential oil in order to obtain a nanobiosystem that could be pelliculised on the surface of catheter pieces, in order to obtain an improved resistance to microbial colonization and biofilm development by *Candida albicans *and *C. tropicalis *clinical strains. The *R. officinalis *essential oils were extracted in a Neo-Clevenger type apparatus, and its chemical composition was settled by GC-MS analysis. Functionalized magnetite nanoparticles of up to 20 nm size had been synthesized by precipitation method adapted for microwave conditions, with oleic acid as surfactant. The catheter pieces were coated with suspended core/shell nanoparticles (Fe_3_O_4_/oleic acid:CHCl_3_), by applying a magnetic field on nanofluid, while the CHCl_3 _diluted essential oil was applied by adsorption in a secondary covering treatment. The fungal adherence ability was investigated in six multiwell plates, in which there have been placed catheters pieces with and without hybrid nanoparticles/essential oil nanobiosystem pellicle, by using culture-based methods and confocal laser scanning microscopy (CLSM). The *R. officinalis *essential oil coated nanoparticles strongly inhibited the adherence ability and biofilm development of the *C. albicans *and *C. tropicalis *tested strains to the catheter surface, as shown by viable cell counts and CLSM examination. Due to the important implications of C*andida *spp. in human pathogenesis, especially in prosthetic devices related infections and the emergence of antifungal tolerance/resistance, using the new core/shell/coated shell based on essential oil of *R. officinalis *to inhibit the fungal adherence could be of a great interest for the biomedical field, opening new directions for the design of film-coated surfaces with antibiofilm properties.

## Background

The increasing occurrence of multidrug resistant, extensive drug resistant and pandrug resistant microbial strains has gradually rendered traditional antimicrobial treatment ineffective [1]. The prognosis is worsened by the formation of microbial biofilms on the biomaterials used in medicine due to their phenotypic resistance, even if the component cells tested in suspension (by the standard method) are susceptible to some antibiotics. Recent public announcements stated that 60% to 85% of all microbial infections involve biofilms developed on natural, intact or damaged tissues (skin, mucosa, endothelial epithelia and teeth), or artificial devices (central venous catheters, peritoneal, urinary catheters, dental materials, cardiac valves, intrauterine contraceptive devices, contact lenses and other implants). The insertion of the prosthetic medical devices for different exploratory or therapeutical purposes, especially in severe pathological conditions, represents a risk factor for the occurrence of chronic infections in developed countries, being characterized by slow onset, middle intensity symptoms, chronic evolution, and resistance to antibiotic treatment [2]. In this context, finding and testing new preventive/therapeutic strategies for prosthetic devices associated infections have become a top priority at the international level. Two main strategies have been employed for the prevention of catheter associated infections: a) development of biomaterials with antiadhesive properties using physico-chemical methods [3-5] and b) incorporation or coating biomaterials which significantly reduce the microbial adherence, e.g., *in vitro *urinary catheters impregnated with silver ions. However, recent research is raising the risk of selecting resistance by coating catheters with antimicrobial substances [6]. Nanotechnology is expected to open some new ways to fight and prevent diseases using atomic scale tailoring of materials [7]. There are a lot of reports on the antimicrobial and antibiofilm properties of different types of nanoparticles, especially heavy metal containing ones (silver, copper, gold and ZnO) ([8-10], as well as the core/shell nanosystems (e.g., CoFe_2_O_4_/oleic acid, Fe_3_O_4_/oleic acid and Fe_3_O_4_/PEG600) [11,12]. Although the potential of natural compounds of vegetal origin to be used as therapeutical remedies is known since longtime, their use is still empirical, but the problem of microbial resistance as well as the negative impact of the chemical substances discharged in the external environment on the ecological balance has reinforced the studies concerning the characterization of the chemical structures of vegetal products and the active doses, aiming to understand their specific mechanisms of action. The efficiency of essential oils obtained from different plant species and their synergic effects as alternative strategies for the treatment of severe infections caused by highly resistant bacteria was already proved [13,14]. The purpose of this study is to combine the unique properties of nanoparticles with the antimicrobial activity of the *Rosmarinus officinalis *essential oil in order to obtain a nanobiosystem that could be pelliculised on the surface of catheter pieces, in order to obtain an improved resistance to microbial colonization and biofilm development.

## Methods

### Extraction and analysis of *R. officinalis *essential oils

The essential oil microwave assisted extraction was performed in a Neo-Clevenger type apparatus, and its chemical composition was settled by GC-MS analysis. Gas chromatographic analysis was performed using an Agilent 6890 Series GC System gas chromatograph (Agilent Technologies Inc., Santa Clara, CA, USA) fitted with a splitless injector for a low background with an injector liner split/splitless under a column head pressure of 12.5 psi and H2 as carrier gas at a flow rate of 1.2 ml/min. Oven temperature was programmed from 50 to 300°Cat 5°C/min. Injector and detector temperatures were 250°C. A capillary column DB5-MS fused-silica J&W Scientific Inc. (Krackeler Scientific, Inc., Albany, NY) (30 m × 0.25 mm i.d.; 0.25 μm film) was used. Detection was carried out with a 5973 mass-selective single quadrupole detector (Agilent technologies). Operation control and the data process were carried out by Agilent Technologies ChemStation software. The mass spectrometer was calibrated before use with perfluorotributylamine as a calibration standard.

### Synthesis and characterization of hybrid nanomaterial core/shell/coated shell

**Figure 1:**
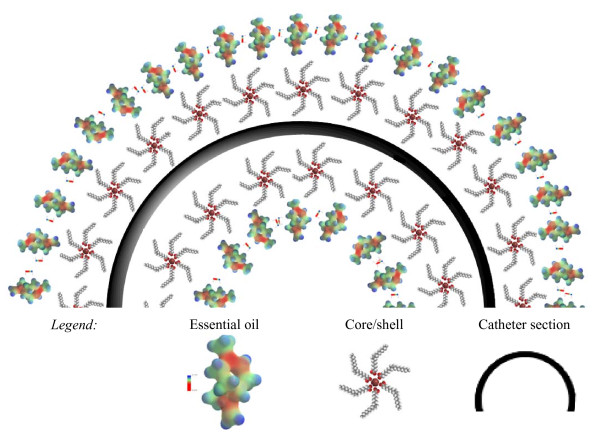
**Core/shell/coated-shell nanobiosystem (transversal section)**.

Functionalized magnetite nanoparticles had been synthesized by precipitation method adapted for microwave conditions, with oleic acid as surfactant. High resolution transmission electron microscopy was used as a primary characterization method for Fe3O4/oleic acid- core/shell nanostructure with dimensions not exceeding 20 nm, according to our previous studies [15,16]. The nanobiosystem was obtained by coating the prosthetic device with suspended core/shell nanoparticles (Fe_3_O_4_/oleic acid:CHCl_3 _0.33% (w/v)). The layer of core/shell nanostructure on prosthetic device was achieved by submerging the catheter pieces in 5 ml of the nanofluid represented by Fe3O4/oleic acid:CHCl_3 _0,33% (w/v). The nanoparticles were aligned in a magnetic field of 100 Kgf applied for 1 s, and thereafter, the catheter pieces have been extemporaneously dried at room temperature. The rapid drying was facilitated by the convenient volatility of chloroform. In order to achieve core/shell/coated-shell type samples, the extra-shell (CHCl3 diluted essential oil, 160 μL/mL) was applied by adsorption in a secondary covering treatment, performed by submerging again for 1 s the catheter samples previously coated with Fe _3_O_4_/oleic acid in 5 ml of CHCl_3 _diluted essential oil, followed by extemporaneous drying at room temperature (Figure 1). The coated prosthetic devices were then sterilized by ultraviolet irradiation for 15 min.

### Fungal model

The artificial monospecific biofilms were developed using two strains of *C. tropicalis *and *C. albicans *recently isolated from clinical specimens and identified by using Vitek II (bioMérieux, Inc., Durham, NC automatic system and previously tested for their susceptibility to currently used antifungals (voriconazole, itraconazole, caspofungin, amphotericin B, fluconazole and flucytosin) and to some essential oils [13,14,17].

### Microbial adherence to the inert and modified prosthetic devices

The microbial adherence ability was investigated in six multiwell plates, in which there have been placed catheters pieces of 1 cm with and without nanobiosystem. Plastic wells were filled with liquid medium, inoculated with 300 μL 0.5 McFarland microbial suspension and incubated for 72 h at 30°C. After 24 h, the culture medium was removed, the catheters were washed three times in phosphate buffered saline (PBS) in order to remove the non-adherent strains, and fresh Glucose broth was added. Also viable cell counts (VCCs) have been achieved for both working variants (coated and uncoated catheter pieces) at each 24 h in order to assess the biofilm forming ability of the two strains. The adhered cells have been removed from the catheter sections by vortexing and brief sonication and serial dilutions ranging from 10^-1 ^ to 10^-^of the obtained inocula have been spotted on Sabouraud agar, incubated for 24 h at 30°C and assessed for VCCs [18].

### Direct examination of biofilm architecture by CLSM

In order to evaluate the biofilm formation on coated and uncoated catheters, a confocal laser scanning microscopy (CLSM) was used. After 48 and 72 h of incubation, the samples were removed from the plastic wells, washed three times with PBS, fixed with cold methanol, and dried before microscopic examination. Samples were visualized in reflection and transmission mode by using a Leica microscope (TCS-SP CSLM model), equipped with PL FLUOTAR (40X NA0.7, electronic zoom 1), and an He-Ne laser tuned on 633 nm wavelength. A lateral resolution of about 600 nm was achieved [19]. The Leica software was used for examining the surface topography.

## Results and discussions

The increasing resistance of *C. albicans *towards the existent antifungal compounds and the reduced number of available drugs led to the search of new therapeutic alternatives among plants and their essential oils, empirically used due to their antifungal proprieties. Plant oils traditionally used for domestic and therapeutic purposes are increasingly claimed to have broad spectrum antimicrobial properties. Some essential oils have been suggested to have potent antimicrobial activity, including antihelmintic, skin infections and insect bites, chicken pox, colds, flu and measles sinus congestion, asthma, bronchitis, pneumonia, tuberculosis, and cholera properties, due to their phenolic, alcoholic, and terpenoid constituents. Agarwal et al. (2008) investigated the inhibitory effect of 30 plant oils against one biofilm forming *C. albicans *strain isolated from a clinical sample, out of which eucalyptus, peppermint, ginger grass, and clove oils proved to be fungistatic, fungicidal, and antibiofilm agents [20].

Previous studies indicated the antimicrobial activity of essential-oil rich fractions of *R. officinalis *against gram-positive (*Staphylococcus aureus *and *Bacillus subtilis*), gram-negative bacterial strains (*Escherichia coli *and *Pseudomonas aeruginosa*) but also against yeasts (*C. albicans*) and molds (*Aspergillus niger*), the main components found in the oil (80%) being represented by alpha-Pinene, 1,8-cineole, camphor, verbenone, and borneol [21].

The identified phytocomponents from *R. officinalis *essential oil analyzed in our study are listed in Table 1. The principal components percentage was 40.596% Eucalyptol, 11.389% Camphor, 10.19% Caryophyllene, and alpha-Pinene 18.42% from the total area.

**Table 1:** Chemical composition of *R.officinalis *L. essential oils

Peak	Compound	RT	Relative content (%)
1	Tricyclene	4.557	0.527

2	Alpha-pinene	4.96	18.42

3	Camphene	5.274	3.532

4	Beta-pinene	6.08	8.162

5	Alpha-phellandrene	6.852	0.164

6	Eucalyptol	7.793	40.596

7	Alpha-terpinene	8.509	0.49

8	Camphor	11.073	11.389

9	Borneol	11.621	0.286

10	Alpha-terpineol	12.394	0.327

11	Bornyl acetate	15.136	2.602

12	Copaene	17.487	0.201

13	Caryophyllene	18.685	10.19

14	Caryophyllene oxide	22.615	0.873

The microbial biofilm is defined as a sessile microbial community composed of cells irreversibly attached to a substratum and between them, embedded in a matrix of extracellular polymeric substances produced by themselves and which presents a modified phenotype with respect to their rate of growth as well as gene transcription [22]. The biofilm cells are resistant to all kinds of antimicrobial substances: antibiotics, antiseptics, disinfectants; this type of resistance, consecutive to biofilm formation is phenotypical, behavioral, and more recently, called tolerance [23]. Tolerance is defined as the ability to survive from bactericidal factors without necessarily expressing a genetic resistance mechanism. The microbial species of clinical interest, often involved in biofilm associated diseases, are belonging to a very large spectrum, from the gram positive (*Staphylococcus epidermidis *and *Staphylococcus aureus*) to the gram negative pathogens (*P*seudomonas *aeruginosa*,and *Escherichia coli*) and to different members of the *Candida *genus. Biofilms formed by fungal organisms are associated with drastically enhanced resistance against most antimicrobial agents, contributing to the persistence of the fungi despite antifungal therapy [20,24].

**Figure 2:**
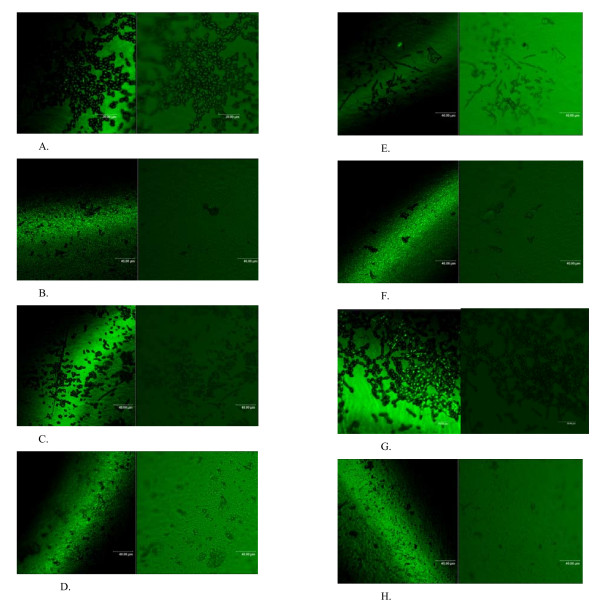
**CLSM images**. (**A**) adherent *C. albicans *strain at 48 h; (**B**) the absence of adherent yeast -cells and *C. albicans *biofilm development in the presence of hybrid nanobiosystem at 72 h; (**C**) adherent *C. albicans *strain at 72 h; (**D**) the absence of adherent yeast-cells and *C. albicans *biofilm development in the presence of hybrid nanobiosystem at 72 h; (**E**) adherent *C. tropicalis *strain at 48 h; (**F**) the rare presence of adherent yeast-cells and *C. tropicalis *biofilm development in the presence of hybrid nanobiosystem at 72 h; (**G**) adherent *C. tropicalis *strain at 72 h; and (**H**) the rare presence of adherent yeast-cells and *C. tropicalis *biofilm development in the presence of hybrid nanobiosystem at 72 h.

Although *C. albicans *is the predominant etiologic agent of candidiasis, other *Candida *species that tend to be less susceptible to the commonly used antifungal drugs such as *C. krusei, C. glabrata, C. lusitaniae*, and the newest *Candida *species, *C dubliniensis*, have emerged as substantial opportunistic pathogens [25]. The outlined work investigates the potential of *R. officinalis *essential oil stabilized in nanolayer and its effect on biofilm formation of *C. albicans *and *C. tropicalis *strains, previously isolated from clinical samples. Confocal laser scanning microscopy (CLSM) was used for the analysis of fungal biofilms developed on the uncoated catheters and on the catheters pelliculised with the plant oils included in the nanosystem. Visualization of control biofilms revealed the ability of the tested strains to form biofilms on the glass coverlisps. The visualization of fungal biofilms developed at 48 h revealed the presence of yeast-phase cells attached to the inert substratum and the microcolony yeast basal layer formation, as well as the expansion of the biofilm architecture by the occurrence of hyphal and pseudohyphal forms, that diminished at 72 h (Figure 2). Visualization of the biofilm structure in the presence of the tested hybrid nanosystem revealed an important reduction in adhering cells and biofilm development as compared to the untreated biofilm, the adhered yeast-cells being practically absent at 48 and 72 h for both tested strains (Figure 2). This suggests that despite the relative minimal diffusion due to the inclusion of the essential oil in the nanoparticles layer, the vegetal compounds may be exerting a metabolic interference in biofilm.

**Figure 3:**
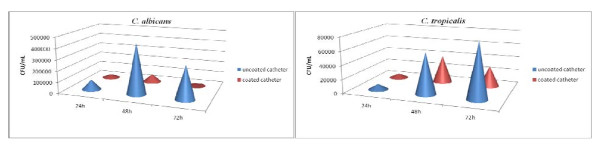
**The viable cell counts of fungal cells**. The viable cell counts of fungal cells adhered and embedded in biofilms formed on the catheter surface (control versus coated catheters).

The antibiofilm effect of the tested nanobiosystem was also proved by the significant reduction of viable cell number in the presence of the protective pellicle of the hybrid nanobiosystem. In the case of *C. albicans *biofilms, although a slight reduction was observed in the early phase of biofilm development (at 24 h), a clear inhibitory effect was observed in the case of 48- and 72-h biofilms, the VCCs drastically dropping with approximately 85% to 98%, as compared to the uncoated controls (Figure 3). Comparatively, in case of *C. tropicalis *biofilm, the most intensive decrease of viable cells adhered to the catheter sections was observed at 24 h (Figure 3). These results could be probably related to the earlier production of pseudohyphae and germ tubes by *C. tropicalis *(48 h) as compared with *C. albicans *(72 h). These structures could represent additional contact and adherence points to inert or cellular substrata and also sources of genetic variability and adaptation to different limitative conditions, being responsible, for example, for the high tolerance to antifungal substances [26].

## Conclusions

The *R. officinalis *essential oil coated nanoparticles strongly inhibited the adherence ability and biofilm development on catheter surface of the *C.albicans *and *C. tropicalis *tested strains, as shown by VCCs and CLSM examination. Due to the important implications of C*andida *spp. in human pathogenesis, especially in prosthetic devices related infections and antifungal tolerance/resistance, using the new core/shell/coated shell based on essential oil of *R. officinalis *to inhibit fungal adherence to prosthetic device could be of a great interest for the biomedical field. These materials-based approaches to control of fungal adherence could provide both (i) new tools to study mechanisms of fungal virulence and biofilm formation, and (ii) approaches to the design of film-coated surfaces or to treat the surfaces of solid and fiber-based materials that prevent or disrupt the formation of fungal biofilms.

## Competing interests

The authors declare that they have no competing interests.

## Authors' contributions

CC conceived the study, provided the fungal strains, and drafted the manuscript together with AG. AG and VG performed the synthesis and characterization of hybrid nanomaterial core/shell/coated shell. CS obtained the essential oil and performed the biological analyses, EA and VL participated in the design of the study and coordination. All authors read and approved the final manuscript.
